# BrainQuake: An Open-Source Python Toolbox for the Stereoelectroencephalography Spatiotemporal Analysis

**DOI:** 10.3389/fninf.2021.773890

**Published:** 2022-01-07

**Authors:** Fang Cai, Kang Wang, Tong Zhao, Haixiang Wang, Wenjing Zhou, Bo Hong

**Affiliations:** ^1^Department of Biomedical Engineering, School of Medicine, Tsinghua University, Beijing, China; ^2^Epilepsy Center, Yuquan Hospital, Tsinghua University, Beijing, China

**Keywords:** epilepsy, stereoelectroencephalography, electrode localization, Epileptogenicity Index, interictal high-frequency oscillation, Hough Transform

## Abstract

Intracranial stereoelectroencephalography (SEEG) is broadly used in the presurgical evaluation of intractable epilepsy, due to its high temporal resolution in neural activity recording and high spatial resolution within suspected epileptogenic zones. Neurosurgeons or technicians face the challenge of conducting a workflow of post-processing operations with the multimodal data (e.g., MRI, CT, and EEG) after the implantation surgery, such as brain surface reconstruction, electrode contact localization, and SEEG data analysis. Several software or toolboxes have been developed to take one or more steps in the workflow but without an end-to-end solution. In this study, we introduced BrainQuake, an open-source Python software for the SEEG spatiotemporal analysis, integrating modules and pipelines in surface reconstruction, electrode localization, seizure onset zone (SOZ) prediction based on ictal and interictal SEEG analysis, and final visualizations, each of which is highly automated with a user-friendly graphical user interface (GUI). BrainQuake also supports remote communications with a public server, which is facilitated with automated and standardized preprocessing pipelines, high-performance computing power, and data curation management to provide a time-saving and compatible platform for neurosurgeons and researchers.

## Introduction

Nearly 30% of the patients with epilepsy eventually become intractable patients resistant to antiepileptic drugs (Kwan and Brodie, 2000). To these patients, the intracranial stereoelectroencephalography (SEEG) surgery, first developed by Talairach and Bancaud at the Hospital Sainte Anne, Paris (Bancaud et al., 1965), is now a common clinical approach to consider about. SEEG aims at identifying the epileptogenic zones (EZs; Rosenow and Lüders, 2001) in the suspicious area of the brain of an individual by implanting depth electrodes and capturing the abnormal neural activities, followed by a resection or thermocoagulation surgery (Cossu et al., 2015; Wang et al., 2020). During this procedure, a large number of neurodata with multiple modalities occur. Presurgical MRI T1 structural image and CT image after the implantation surgery can, respectively, be taken as information for brain surface reconstruction and SEEG electrode localization (Behrens et al., 1994; Dykstra et al., 2012). Neural activities before the resection surgery are recorded with SEEG electrodes for EZ localization and lesion analysis, usually lasting for 2 weeks. The neural activity acquired during the 2-week SEEG recording is vital to the presurgical planning (Cossu et al., 2015) and of great value to brain research (Zhang et al., 2019; Akkol et al., 2021). However, exploiting the large number of multimodal neurodata and managing them effectively remain a problem to be solved.

The SEEG electrode localization procedure using co-registered MR and CT images provides neurosurgeons with accurate anatomical positions of the implanted electrode contacts (Dykstra et al., 2012). The traditional and broadly used method of electrode contact localization mostly depends on visual checking and manual operations (Darcey and Roberts, 2010). After the registration of MR and CT images, technicians view the CT image slice by slice, locating highlighted contact voxels and mapping the positions onto the MRI (Darcey and Roberts, 2010). Trouble occurs since every patient may have 100 contacts implanted on average, and one should check the slices back and forth for a highlighted contact centroid, which is a complicated and time-consuming task. Several previous studies have proposed semiautomated methods (Blenkmann et al., 2017; Hamilton et al., 2017; Narizzano et al., 2017; Qin et al., 2017; Li et al., 2019) to improve the effectiveness and precision of electrode contact localization. The SEEG Assistant (SEEGA) extension of the 3D Slicer applies an algorithm of the center-of-mass convergence for the contact segmentation step (Arnulfo et al., 2015; Narizzano et al., 2017), which shows great feasibility and robustness in locating contacts along each electrode shaft. However, this method requires a prior manually defined fiducial file of the planned starting and ending points of each electrode and an additional presurgical CT scanning. Another study (Qin et al., 2017) inherits the convergence algorithm and develops a preprocessing workflow to reduce the required input. This workflow includes MRI and CT registration, masking, eroding, and clustering steps but still needs to insert several pause points for visual checking and manual adjustments. Another toolbox (Blenkmann et al., 2017) implements a k-means clustering algorithm to segment contacts along each electrode, in which the voxels of each electrode should be carefully thresholded, otherwise the contacts may not be completely segmented.

In the clinical SEEG data analysis, doctors are mainly concerned about the effect of a few episodes of ictal data for the location of EZs. Channels with relatively early abnormal activity during the seizure often indicate the potential EZs. A previous study defined an Epileptogenicity Index (EI) using the onset of high-frequency energy to predict the onset area (Bartolomei et al., 2008). However, in some cases, the onset period may not be captured to provide sufficient diagnostic information. In contrast to only a few seizures during the monitoring period, most of the SEEG signals recorded are seemingly ordinary interictal data. The sporadic abnormal activities in the interictal interval, such as spikes or high-frequency oscillations (HFOs), can be used as plausible pathological markers of EZs. Because the intracranial EEG recording consumes huge storage space, recording an 80-channel intracranial EEG at a sampling rate of 2,000 Hz for 24 h may generate a data volume of about 50 GB. It is time-consuming for surgeons to extract sparse interictal pathological activities from the long-term SEEG. Currently, the interictal data cannot be fully and effectively traversed by surgeons and thus is usually deleted. The value of the interictal data is mostly underestimated. Therefore, there is an urgent need to detect abnormal activities in interictal SEEG data to extract pathological information and reduce the workload of clinicians. Both HFO activities (Navarrete et al., 2016) and spike detection algorithms (Barkmeier et al., 2012) have been developed based on waveform morphology, but indexation methods that efficiently extract interictal epileptic discharge events are yet to be developed. In addition, the performance of current interictal event detection methods heavily depends on the manual selection of the parameters (Remakanthakurup Sindhu et al., 2020). Our interictal data analysis module is designed to minimize manual interference by implementing an automatic HFO detection method and retaining only necessary parameter settings such as filter ranges and channel selections.

After electrode localization and data analysis, cortical surface reconstruction is an essential step for better visualization. Several previous studies have developed the reconstruction procedure (Dale et al., 1999; Fischl, 2012; Henschel et al., 2020; Zöllei et al., 2020). FreeSurfer group releases tools and pipelines publicly (Fischl, 2012). They built a reconstruction pipeline, “recon-all,” covering from primary operations such as motion correction and skull-stripping, to final steps such as segmentation and cortical parcellation. Several subsequent studies have also proposed advanced reconstruction tools such as specifically, “infant-FreeSurfer” (Zöllei et al., 2020) for covering all ages of subjects and “FastSurfer” deep learning pipeline (Henschel et al., 2020) for solving the time-consuming problem. However, FreeSurfer software and its advanced tools can only be executed on Linux-based operating systems (OS). Virtual machine configuration and the usage of terminal lines can be troublesome for some Windows users. Moreover, there is often a lack of local computing power for rapid surface reconstruction in the clinical setting.

In this study, we present BrainQuake, an open-source Python software, providing epilepsy surgeons with tools and integrated pipelines of surface reconstruction, electrode contact localization, and ictal and interictal SEEG analysis for presurgical evaluations. The integration aims at automatically executing the whole workflow with fewer input files and fewer pause points. BrainQuake is designed as an end-to-end, highly automated, time-saving software, free to be downloaded and compatible with both Linux and Windows OS. With a comprehensive data processing platform established, surgeons can take the most advantage of neurodata and make reliable presurgical evaluations for those epilepsy patients. We hope this software can be helpful to clinical practice and human neuroscience studies using SEEG.

## Materials and Requirements

### Software Overview

**Figure 1:**
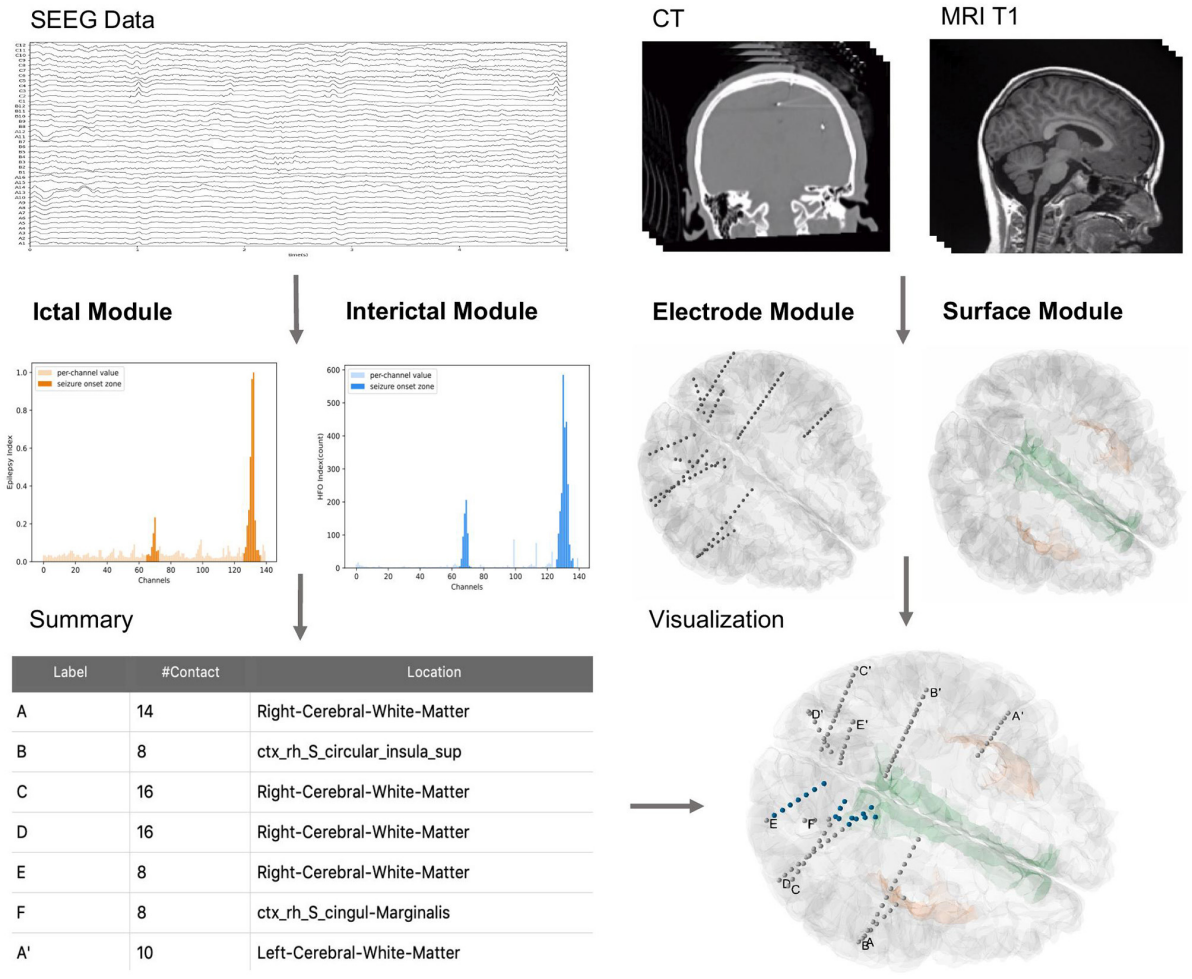
General overview of BrainQuake structure. BrainQuake is designed to analyze the SEEG data (ictal and interictal), CT images, and MRI T1 images. Ictal and interictal modules are used to predict suspect contacts within seizure onset zones (SOZs). The electrode module exploits the graphic information from a CT image to locate the stereoelectroencephalography (SEEG) electrodes and contacts, as well as project them onto the brain surface, which is reconstructed by the surface module. The locations of suspect contacts are marked (blue) on the 3D plot of the surface and electrodes, giving a brief overview of the presurgical evaluation results.

**Figure 2:**
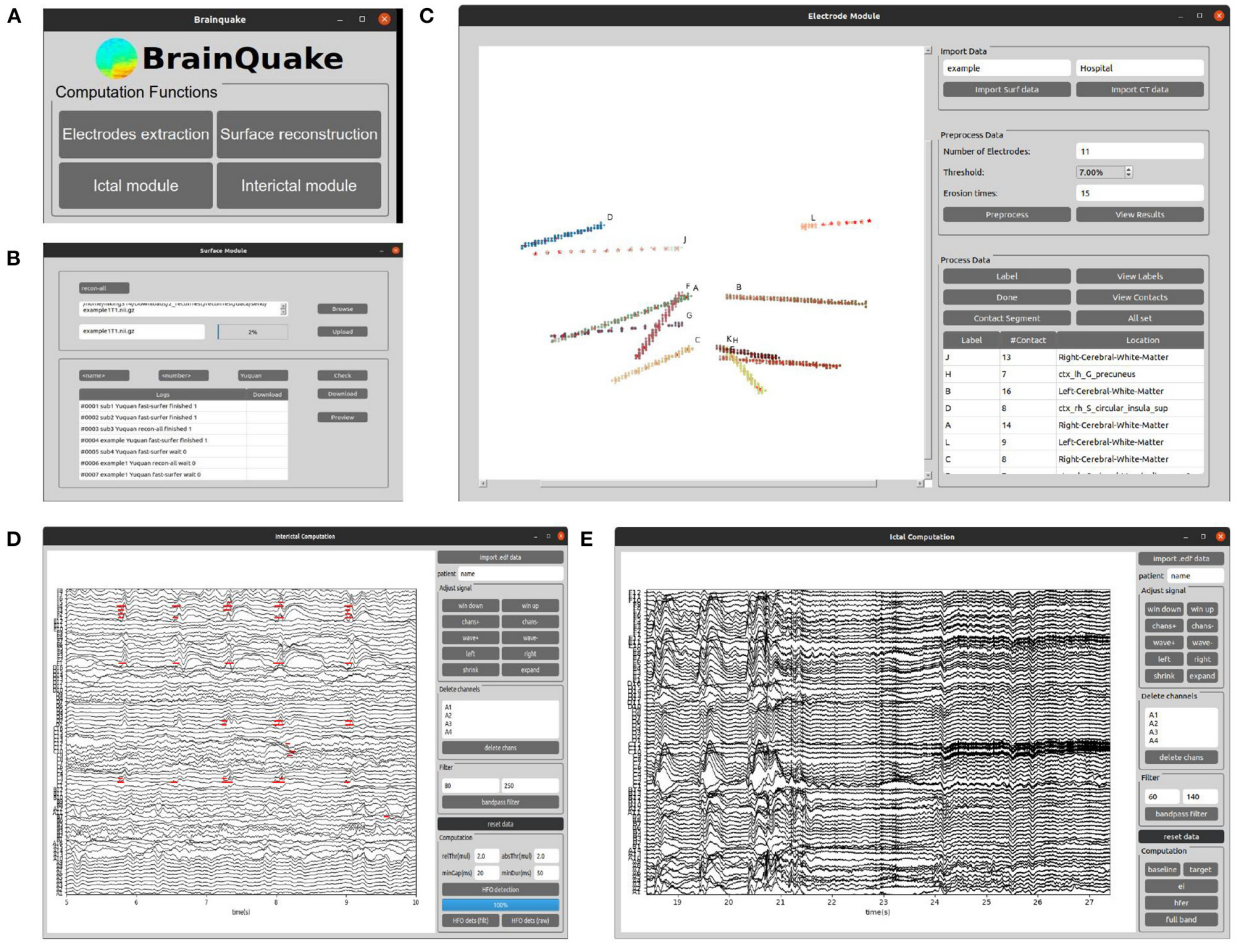
The graphical user interfaces (GUIs) of the main window of BrainQuake and the four functional submodules. **(A)** BrainQuake main window; **(B)** Surface module; **(C)** Electrode module; **(D)** Interictal module; **(E)** Ictal module.

BrainQuake is an open-source Python software for image and SEEG data processing of refractory epilepsy patients. BrainQuake consists of four modules, namely, surface module, electrode module, ictal module, and interictal modules (Figure 1). The surface module is used for surface reconstruction of the MRI T1 image of the patient. We incorporated a GUI, a client-server communication mode, a public server with powerful graphics processing units (GPUs), and a data curation system, to ensure that users share a time-saving, private, and stable data preprocessing pipeline. The electrode module consists of a pipeline to locate and anatomically label the SEEG electrode contacts using both preoperative T1 image and postoperative CT image. The ictal module and interictal module analyzed the recorded SEEG data and then pinpoint the suspicious seizure onset zones (SOZs) using EI and High-Frequency Events Index (HI), respectively. Finally, BrainQuake provides a comprehensive visualization result of the 3D brain surface of an individualized patient, with SEEG contacts and SOZ predictions projected on it. We developed GUIs for all these modules (Figure 2), and tutorials can be found along with installation packages.

### Data

#### Subjects

The SEEG electrodes, or intracranial depth electrodes, were used in human subjects undergoing epilepsy surgical treatment. We analyzed the data from five patients temporarily implanted with SEEG electrodes (8–16 contacts per electrode, 2 mm diameter, and 3.5-mm center-to-center spacing). Intracranial EEG was continuously recorded for 2 weeks on average, and MRI and CT images were, respectively, acquired before and after the implantation operation. The surgeries were conducted in the Department of Neurosurgery and Epilepsy Center, Tsinghua Yuquan Hospital. Data collection and scientific workup were approved by its Institutional Review Board.

#### Example Data

**Figure 3:**
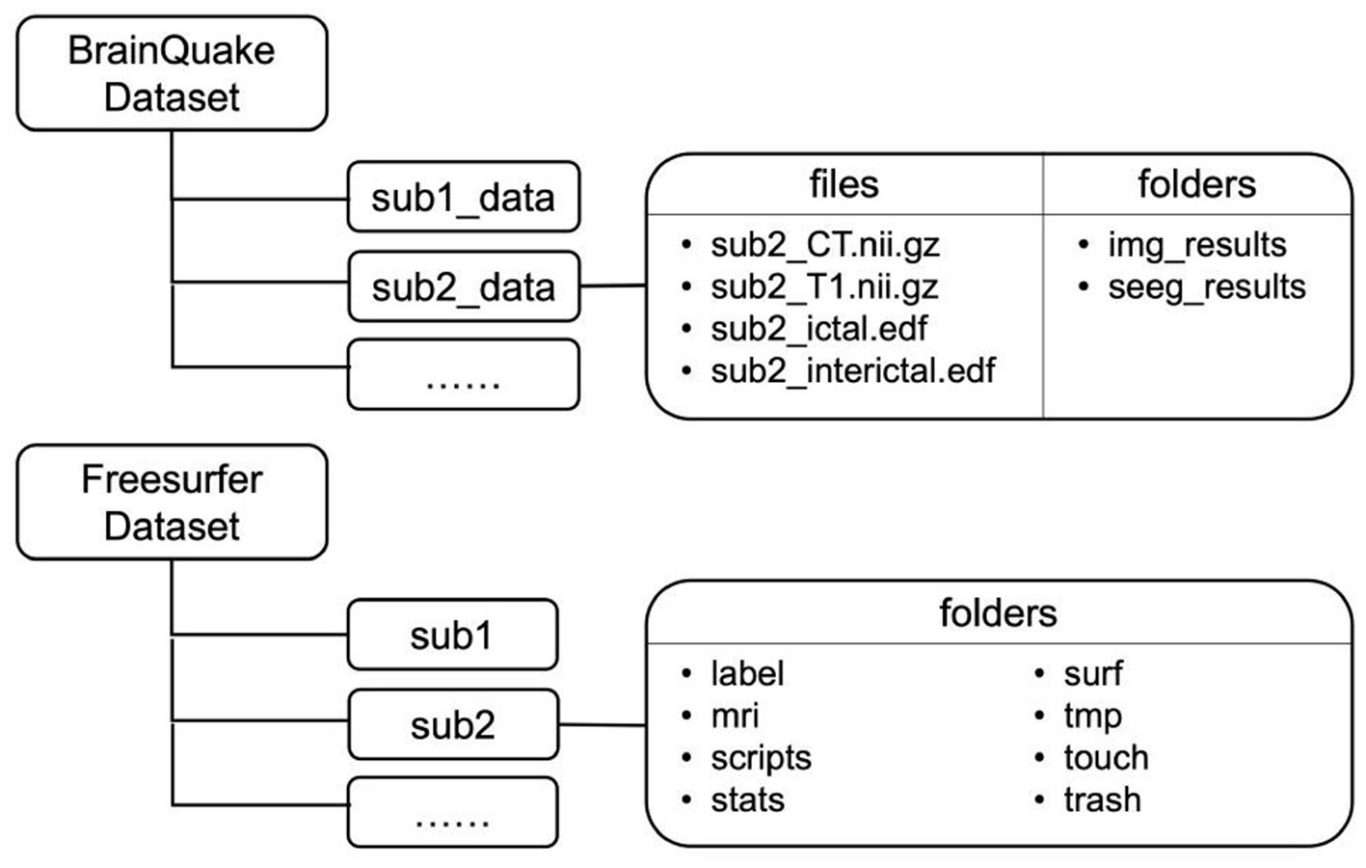
File structures of two datasets implemented in BrainQuake. Temporary and final results are saved under the folders of each subject.

We provided eight sets of sample data so that potential users can follow the data format and file structure and go through the procedures in BrainQuake. Sample data are available at https://doi.org/10.5281/zenodo.5675459, such as MRI T1 image, CT image in NIfTI-1 type, and recordings of ictal and interictal EEG data **(**up to 2 h per patient) for each sample. The file structure is shown in Figure 3. FreeSurfer “recon-all” results are also included since we used some of their intermediate files (mri/orig.mgz, brainmask.mgz; surf/lh.pial, rh.pial) in our modules. Two separate directories, namely, BrainQuake dataset and FreeSurfer dataset, will be configured during the initialization of the software.

#### Operating Requirements

The codes are divided into the client part and the server part. Computers running either Linux, Mac OS X, or Windows should run the client Python GUI code. For the server part, it should be running on Linux or Mac OS X, since FreeSurfer works only on Linux. We recommended users install the client GUI code and communicate with a public server we provided and leave all the time-consuming works (e.g., surface reconstruction, CT and MRI image registration) to it. Essential processed data for functional modules in BrainQuake can be downloaded from the server. If facilitated with a Linux-based server at local, one can still download and install the server codes and run the whole pipeline within their own workspace. On the remote server side, FreeSurfer (version 6 or higher) should be properly installed as well as the packages mentioned previously. Full installation tutorials can be found on https://github.com/HongLabTHU/Brainquake. Detailed operating requirements are listed as follows:

1. Computers running on Linux, Mac OS X, and Windows should run the client codes (i.e., Python scripts outside the “Server_codes” folder on the GitHub of BrainQuake).
2. Server codes should be run on a Linux-based server, with FreeSurfer (version 6 or higher) installed.
3. Processor speed: 2.0 GHz or higher recommended.
4. RAM: 8 GB or higher recommended.
5. Python version: 3.6 or higher.
6. Third-party dependencies: numpy, nibabel, matplotlib, scikit-learn, scipy, mne, vtk, and mayavi.

The public server [Ubuntu 18.04, 40 central processing units (CPUs), 2.10 GHz] we provided assigns eight cores to each “recon-all” task for parallel computing and can hold up to three tasks running simultaneously. Each “recon-all” task lasts 3 h on average. Server codes are also provided on the GitHub of BrainQuake so that one can facilitate their own server for reference. The output package of a surface reconstruction task from the server pipeline of BrainQuake includes a typical reconstruction result folder (produced by FreeSurfer), an “orig.nii.gz” file (produced by FreeSurfer command “mri_convert”), a “mask.mgz” file (produced by FreeSurfer command “mri_binarize”), and a registered “ <name>_CT_Reg.nii.gz” file (produced by FSL command “flirt” with “orig.nii.gz” as its reference image). Producing all of these files and folders requires FreeSurfer installed in the operating environment, so if a potential user prefers not to apply the client-server mode, one can always import their own “recon-all” folders with all these Supplementary Files prepared.

## Methods

### Image Processing Modules

#### Surface Module

FreeSurfer provides a complete pipeline, “recon-all,” for surface reconstruction, which is compiled with abundant tools such as skull-stripping, image registration, cortical reconstruction, and segmentation. More time-saving or specific pipelines such as “FastSurfer” (Henschel et al., 2020) and “infant-FreeSurfer” (Zöllei et al., 2020) have been released in recent years. We integrated all those pipelines in the provided server and also provided processing options in the surface module GUI so that users no longer need to deal with the terminal when using “recon-all” or wait too long for a reconstruction result since the server is facilitated with GPUs and the average processing time is 3.5 h for “recon-all” and only 30 min for “FastSurfer” and “infant-FreeSurfer.” Windows users need not configure a virtual machine for installing FreeSurfer locally since our server can undertake all the preprocessing works.

#### Electrode Module

**Figure 4:**
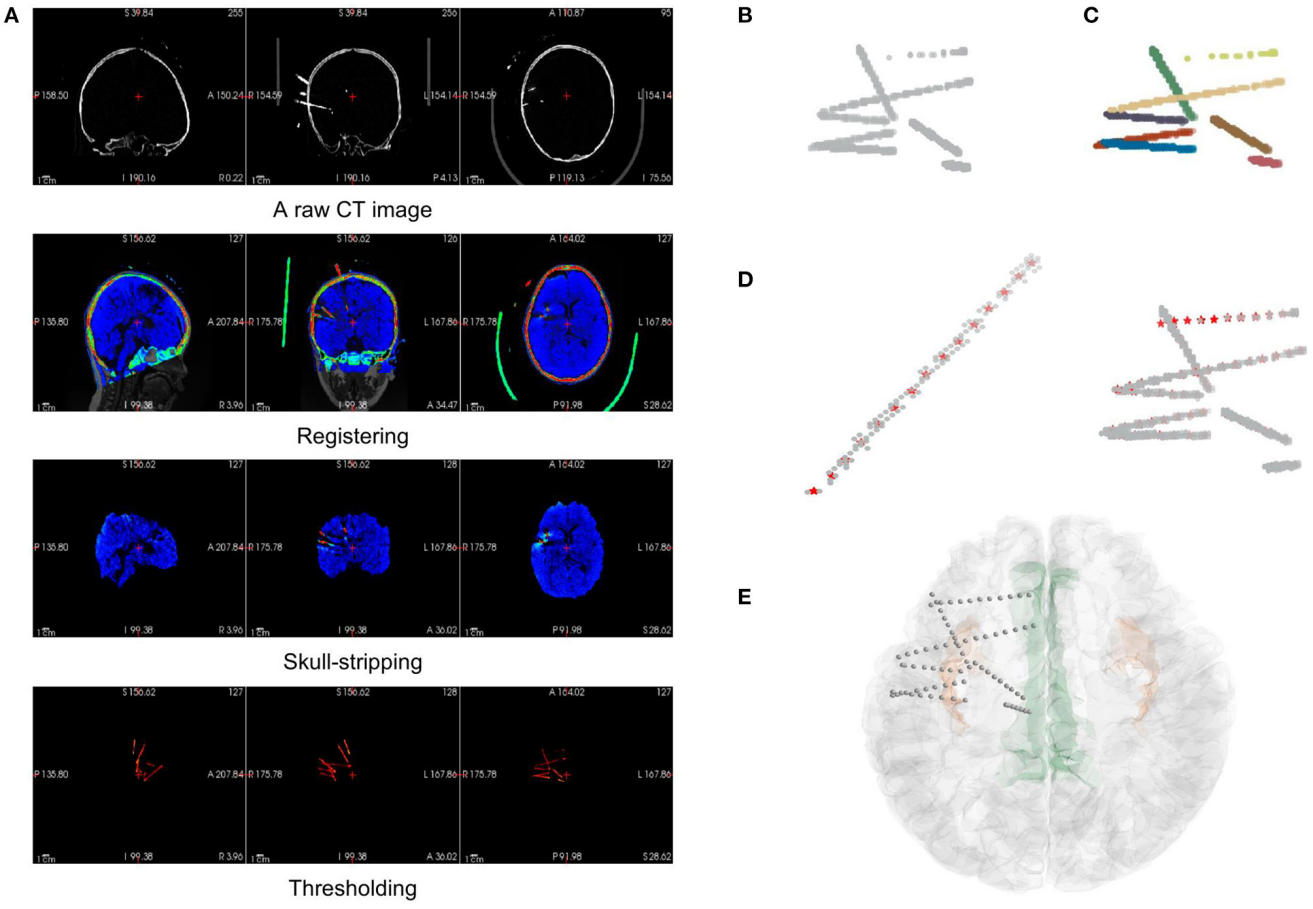
The pipeline of electrode localization and contact segmentation procedures in the electrode module. **(A)** The preprocessing step includes image registration from the raw CT of a subject to MRI (orig.mgz after surface reconstruction), skull-stripping of registered CT (using brainmask.mgz after surface reconstruction), and thresholding of electrodes in the CT data. **(B)** The coordinates of electrode voxels in the CT image after thresholding can be extracted and plotted, viewing as a mix of point clouds. **(C)** After applying a Hough Transform and Gaussian Mixture Model algorithm, the electrodes are clustered and labeled by different colors. **(D)** Contact segmentation step: contact positions are recognized one by one by converging to the center-of-mass based on voxel values. Contact positions are marked as red asterisks. **(E)** The results of the contact segmentation pipeline are projected onto the 3D surface space.

Either processed manually or semiautomatically, the main idea of electrode contact segmentation is to identify the brightest voxels in a CT image as contact positions along each depth electrode. To conduct an autonomous pipeline of contact segmentation, we should make the best use of the image properties. The electrode module of BrainQuake requires the input data of only a postsurgical CT NIfTI image and a result package of surface reconstruction. The pipeline in the module includes three parts, namely, image preprocessing, electrode clustering, and contact recognition (Figure 4).

##### Preprocessing

Before we could autonomously identify an electrode or contact, we must ensure that the image contains only the intracranial area of a brain since the skulls, teeth, or some electrode supports outside the brain are hard to be distinguished from the electrodes based on the voxel value difference of a CT image. In the preprocessing step, we registered the CT with the standardized MR image generated in the surface module. This registration step uses FSL “flirt” (Jenkinson et al., 2012) after surface reconstruction in the surface module. Then, the registered CT can be masked with a skull-stripped MR image in the surface data package to remove the extracranial part of the CT data since they are now in the same coordinate. At this time, the CT image contains only the information about the intracranial brain and the electrodes, the two of which show a significant difference in their voxel value ranges. Electrode voxels are much brighter in the image, so they can be extracted simply by thresholding (Figure 4A).

##### Hough Transform and Gaussian Mixture Model

**Figure 5:**
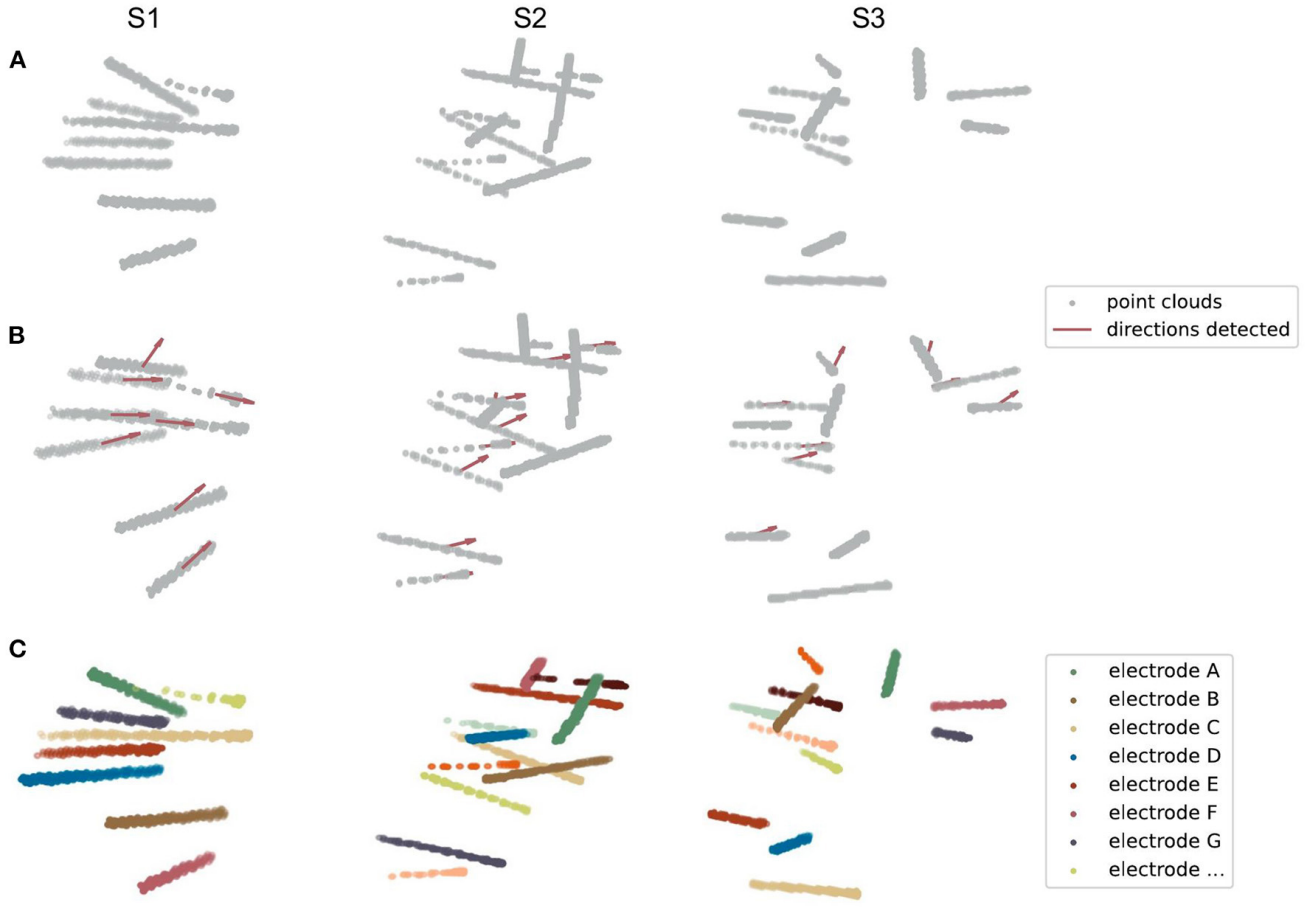
Three examples of electrode point clouds have been 3D Hough-transformed and then clustered using the Gaussian Mixture Model. **(A)** The initial point clouds of electrodes are extracted from the CT intracranial image of an individual after several preprocessing steps. **(B)** The centroids and directions (showing by the red arrows) of SEEG electrodes are detected by the Hough Transform algorithm of a line in 3D coordinates. **(C)** The clustered electrodes are marked using different colors, after applying the Gaussian Mixture Model and the prior knowledge of the centroids and directions of clusters generated from **(B)**.

After extracting the electrode voxels into point clouds (Figure 4B), we need to identify the number and axes of electrodes and label each voxel into different electrode clusters. This step is completed in most of the previous works by clustering algorithm with manual adjustment. In BrainQuake, we developed a method of combining 3D Hough Transform, a pattern recognition algorithm, and Gaussian Mixture Model, a clustering algorithm, to label voxels into different electrode clusters (Figure 5).

Normal clustering algorithms randomly pick some centroids in CT images, classify the voxels into clusters, and calculate the new centroid of each cluster. After multiple iterations, theoretically, voxels belonging to the same electrode can be assigned to the same cluster. However, the clustering algorithm is strongly dependent on the initial selection of centroids. With an improper initialization of the random centroids, the true distribution of electrode clusters can be difficult to estimate. There is a high probability that we would get a locally optimal clustering result, definitely requiring a manual intervention here to fix it, for example, to merge some of the clusters to form a real electrode or to split two or more electrodes in the same cluster.

Our method fixes this issue by adding a Hough Transform before clustering. Hough Transform is a common method used in computer vision or digital image processing (Illingworth and Kittler, 1988). It can be used to detect a certain class of shapes in an image automatically. The main idea of Hough Transform is that for a specific shape, we have chosen a set of parameters and created a parameter space. For example, the parameter we usually used to describe circles can be center and diameter, while the parameter of 2D lines can be slope and intercept. Suppose we have a raw image with a mixture of dots on it. Each dot will vote in the parameter space for every possible parameter set they can contribute. Positions in the space with the highest votes are recognized as the parameter sets describing the most obvious shape in the raw image. In our case, SEEG electrodes in a CT image are a combination of line-shaped objects in 3D space. The parameter space is established to represent the line direction (horizontal orientation and altitude) and the distance between the coordinate origin and the line.

First, we transformed those voxels into point clouds (Figure 5A). Then, we applied a 3D line Hough Transform to detect line-shaped trajectories (Jeltsch et al., 2016; Dalitz et al., 2017) in the point clouds, returning centroid and axis direction of each electrode cluster. At this stage, we got a set of approximate but not precise results representing the position of each cluster (Figure 5B), which can be a good set of prior knowledge to start clustering. After that, we used the Gaussian Mixture Model (Reynolds, 2009; Pedregosa et al., 2011) to assign each point to the electrode cluster it belongs, since the point clouds can be viewed as a mixture of different line-shaped 3D Gaussian kernels (Figure 5C). After a successful clustering, the axes directions of electrodes can be regressed (Pedregosa et al., 2011). This combinatory method makes use of both electrode geometric prior and voxel distribution in a CT image, which shows excellent accuracy and robustness in our experiments.

##### Contact Segmentation

In the SEEG contact segmentation step, our general goal was to automatically recognize the relatively brightest voxels, which are viewed as contact positions, along each electrode shaft. We mainly divided the process into four sub-steps, namely, locating the head voxel, locating the target contact, stepping toward the next contact, and locating the rest contacts along the shaft.

First, we applied a linear regression (Pedregosa et al., 2011) to each electrode cluster of voxels to get the direction parameter (coefficients between x-y/y-z/z-x axes) of the electrode shaft track in the 3D space coordinate. We then used the direction to locate two voxels, respectively, to be the head and tail of the cluster. As a general assumption that the head voxel is always closer to the center of the brain (i.e., the center of the image space), we can locate the position of the head voxel, which is much close to the target contact.

Second, we applied a “center-of-mass” convergence algorithm (Arnulfo et al., 2015) to locate the target contact. We viewed each voxel value as the “mass” of a single voxel or “weight” of this point. In this way, the center-of-mass is defined as the “heaviest” point within a small region of voxels. After finding out the head voxel, we calculated the center-of-mass of its surrounding region (a geometry-restricted cubic volume with respect to the actual contact size, 2 × 2 × 2 mm cube in our case). We then again calculated the next center-of-mass within the surroundings of the newly found center-of-mass. After 1–2 iterations of this procedure, the calculated center-of-mass eventually converges to the brightest voxel around the head of the electrode (i.e., the real target contact position).

Third, as we already knew the electrode track direction and the target contact, stepping out a specific distance along the direction from the target contact can give us a position close to the next contact. The step size should equal the real distance between two adjacent contacts (3.5 mm in our case). In this case, we made sure that the position found was close enough to the next contact, which was ready for another center-of-mass convergence procedure.

Finally, using the same center-of-mass convergence and the stepping strategy, the rest contacts can be recognized one by one. In this iterative process, we also set a geometrical restriction to ensure that the directed positions are always settled within the cluster by doubling the weights of the voxels in the cluster (Figure 4D).

##### Validation Method of Electrode Localization

We used two methods to validate the results of the electrode module, namely, visual inspection of the electrode positions and quantitative measurements of the electrode contact distribution. The recognized contacts were projected onto the 2D slice of the fusion of MR and CT images. Then, we scanned through all these slices and visually checked if the electrodes and the highlighted electrode shaft on CT slices were overlapped.

To quantitatively estimate the accuracy of contact localization, we must define a gold standard of contact positions and then estimate the contact deviation error one by one. Usually, a group of clinical experts should be invited to view through all those image slices and mark the contact positions manually. However, due to the artifacts of each contact in the CT images, one may find it tough to segment those contacts since the adjacent contact pairs are usually merged. Thus, we could not trust the manual segmentation results as a gold standard. In this study, we estimated two indirect metrics, namely, axis-contact distance (i.e., distances between contacts and their estimated shaft axis) and adjacent contact distance of each adjacent contact pair (Arnulfo et al., 2015; Narizzano et al., 2017). Both of the metrics are based on the geometric properties of the SEEG electrodes. Contacts along the same electrode shaft are line-shaped regressed, and the axis-contact distance ideally can be close to 0 mm. The axis-contact distance is defined as the distance between the contact position and the regression line of the electrode shaft. It reveals how straight the contacts are located. The electrodes we used have a fixed spacing distance of 3.5 mm between neighboring contacts, so the adjacent contact distance we estimated should be distributed similarly to a Gaussian with a mean of 3.5 mm and a trivial variance as much as possible. However, it is often the case that the electrode shaft bends slightly and the contacts deviate from the line after the implantation surgery, which in some way causes these two distributions to be not so ideal (refer to the “Discussion” section).

### SEEG Data Analysis Modules

#### Ictal Module

For ictal data, clinicians tend to mark the areas where the pathological activity occurs earlier as the potential SOZs. Based on this consensus, an EI method is commonly used to predict the SOZs (Bartolomei et al., 2008). In this study, we implemented a simplified EI measurement in BrainQuake, predicting the SOZs by quantifying the combined effect of the timing order and the strength of high-gamma energy change in each channel during the onset process of the seizure (Zhao et al., 2019).

Before we did any automatic computation, we first filtered the raw signals into high-gamma frequency bands (60–140 Hz, power noise at 50 Hz) using a second-order IIR notch digital filter and a fifth-order Butterworth IIR filter (Virtanen et al., 2020). We then manually selected a segment of the baseline (BL) data, as well as a segment of the target data containing the initial onset process of seizure. The BL data should be located before the seizure onset, and a range of around 60 s should be enough for it. The target data should cover the seizure onset process, that is, to start somewhere before the onset and end within the seizure. The length of the target data is not limited as long as it covers the seizure onset process.

**Figure 6:**
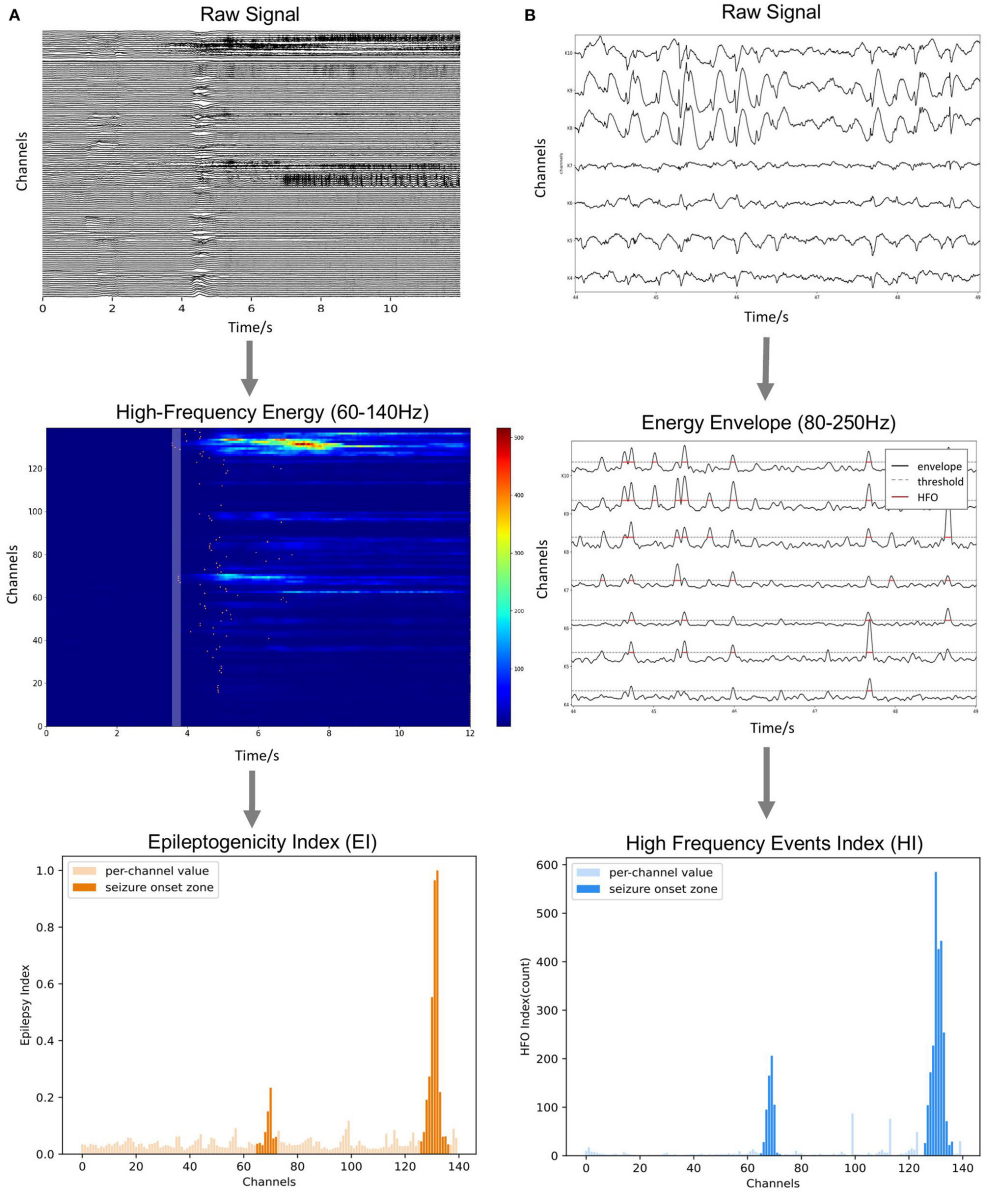
Methods of ictal and interictal SEEG data analysis. **(A)** Onset timing order and energy strength during the initial stage of seizures are sorted to calculate the Epileptogenicity Index (EI). **(B)** Numbers of over-threshold high-frequency events are counted as High-Frequency Events Index (HI).

After the manual selection, we calculated an EI for each channel. First, the band-passed signals are transformed into a high-frequency energy spectrum by amplitude squaring and window smoothing (500-ms window length, 1 sample point per step). Second, we calculated the average value of the high-frequency energy of the BL data, which is used to normalize the high-frequency energy by division. In this way, we obtained the normalized high-frequency energy (NHFE) (Figure 6A). Third, a threshold of onset time was calculated for each channel *i*, which is 10 times the standard deviation (SD) of baseline (BL) NHFE above its maximum value as follows:


$$\begin{array}{l} thre_{i}=\max\left(NHFE_{BL,\;i}\right)+10\sigma \left(NHFE_{BL,\;i}\right) \end{array}$$

For each channel, once the normalized energy in the target data exceeds its corresponding threshold, we decided this moment as the onset time of its abnormal activity. Fourth, we sorted the channels by their onset time and defined the time coefficient (TC) as the reciprocal of the order of each channel (i.e., 1, 1/2, and 1/3). Also, we calculated the average energy of each channel in a 250-ms period right after the earliest onset time as energy coefficient (EC) using the NHFE. Finally, the EI of each channel *i* is obtained by the following:


$$\begin{array}{l} EI_{i}=\sqrt{TC_{i}\times EC_{i}} \end{array}$$

As we can notice, EI, combining the effect of timing and energy strength, can be used to quantify the degree of epileptogenicity of each electrode channel (Figure 6A).

#### Interictal Module

A previous study on the SEEG interictal data found that both HFOs and spikes are the reliable biomarkers of SOZ, while HFO has better specificity for SOZ than spikes (Wang et al., 2017; Roehri and Bartolomei, 2019). The HFO subcategory, 80–250 Hz ripple component, is relatively more common than a higher frequency component (Wang et al., 2013). This frequency band can also take into account the spike activity, which is similar to a full-band signal (Roehri et al., 2017; Cai et al., 2021). Therefore, for the interictal data, we extracted the pathological activity by detecting the short-term abnormal energy enhancement in the 80–250 Hz band, providing an efficient indexation method through unified energy detection. Specifically, we used the Hilbert transform to extract the energy envelope in the 80–250 Hz band of the signal (i.e., users can adjust the frequency range for their own cases). The filter setting applied is a second-order IIR notch digital filter with a quality factor set to be 30, followed by a five-order Butterworth band-pass filter (Virtanen et al., 2020). We calculated the median value of the whole envelope (global, *S*_*global*_) and the median value of each contact (local, *S*_*i*_). Considering both of them, we set a synergistic threshold for each contact as follows:


$$\begin{array}{l} thre_{i}=2\times \max\left(median\left(S_{i}\right),\;median\left(S_{global}\right)\right) \end{array}$$

The time range where the envelop exceeds the threshold is marked as abnormal activity (Figure 6B). When the interval between two adjacent abnormal activities is too small (<20 ms), they are considered to belong to the same event and merged, and the abnormal activities of the very short duration (<50 ms) are excluded. Finally, the number of abnormal activities (HI) calculated for each channel is used as an index to measure the relative likelihood of each contact of being in the SOZ.

## Results and Validation

We processed all four functional modules using the MRI/CT images and the SEEG data acquired from 8 epilepsy patients. The time required for surface reconstruction was either around 0.5 h using FastSurfer or 3.5 h using FreeSurfer recon-all on the public server (40 cores, 2.1 GHz, 64 GB RAM). The preprocessing step in the electrode module for each subject is around 15 min, mostly spent on the image registrations of MRI and CT using the FSL “flirt” command. Contact localization consumes only 30 s for each subject on average. A 70-s interictal SEEG costs around 40 s for EI calculation, and the 2-h interictal data costs around 20 min for HI calculation.

### Electrode Module Validation

**Figure 7:**
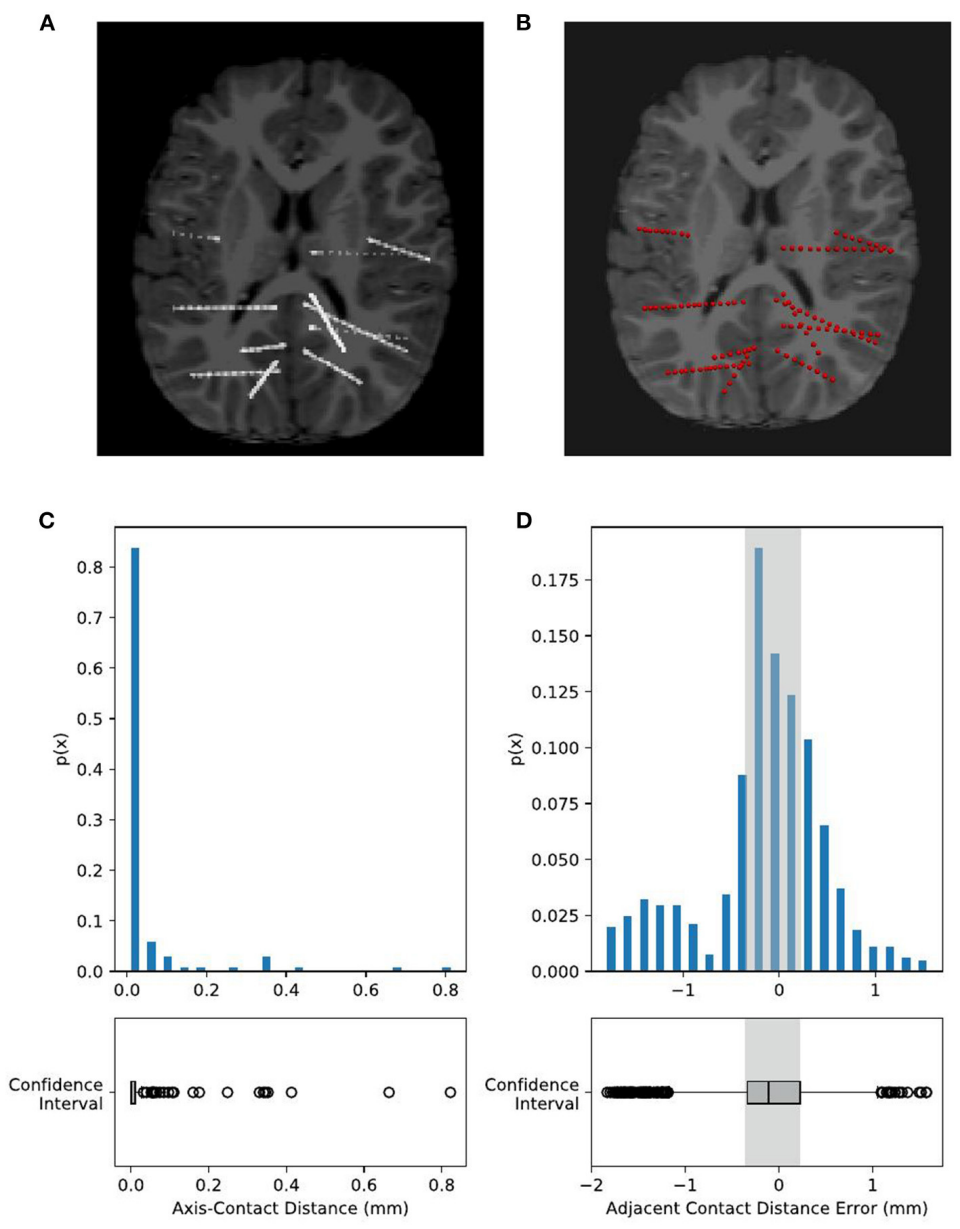
Validation of electrode localization accuracy. Visual checking of the electrodes and contacts of an example subject projected onto the CT image of an individual. The raw CT brain **(A)** shows electrode positions as highlighted line-shaped voxels. Our recognized electrodes (red spheres) are plotted on **(B)**, showing that they are overlapped with each other. Contact positions are quantitatively estimated by two metrics, namely, axis-contact distance and adjacent contact distance error. **(C)** Axis-contact distance estimates the distribution of deviation distance between each contact and its regressed electrode shaft line. Of note, 95% of the contacts were less than 0.1 mm, deviating from their estimated shaft line. **(D)** Adjacent inter-contact distance error estimates the distribution of the distance between each pair of adjacent contacts. The actual adjacent contact distance size, 3.5 mm, is subtracted from the estimated distances, so here we have shown the distribution of the adjacent contact distance error. Notably, 95% of the contact distance fell in the range of 0 ± 1 mm, and 50% of the contact distance fell in the range of 0 ± 0.3 mm, i.e., the adjacent contact distance distribution is 3.5 ± 1 mm (95%) and 3.5 ± 0.3 mm (50%).

We processed 74 electrodes with 743 contacts implanted in eight patients in total. During visual inspection, all 74 electrodes were perfectly matched with the highlighted electrode shaft artifacts on CT images (Figures 7A,B). For quantitative validation, we estimated two metrics, namely, axis-contact distance and adjacent contact distance error, to measure whether the distributions of recognized contacts obey the geometric rules of the SEEG electrode. In statistics, 95% of the contacts were <0.1 mm, deviating from their estimated axes (Figure 7C). By the subtraction of 3.5 mm (real adjacent contact distance) mean, the adjacent contact distance error was distributed around 0 mm with a Gaussian-like distribution. Notably, 95% of the contact distance fell in the range of 3.5 ± 1 mm, and 50% of the contact distance fell in the range of 3.5 ± 0.3 mm (Figure 7D). These two estimates show comparable results with the Contact Position Estimator (CPE) Module of 3D Slicer (Narizzano et al., 2017).

### SEEG Analysis Validation

**Figure 8:**
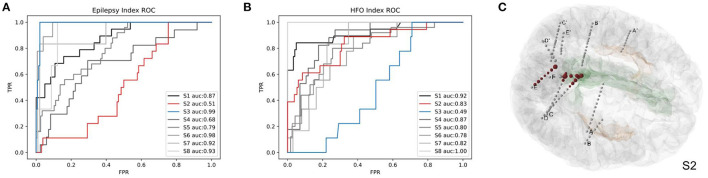
Validations of the SOZ prediction results of BrainQuake comparing with clinically annotated SOZs. **(A)** The receiver operator curve (ROC) and area under the curve (AUC) results of SOZ prediction, based on EI. Case S2 shows a low AUC of 0.51 (low predictive effect), while HI-guided prediction of S2 is 0.83, which is highly consistent with the clinically annotated SOZs. That is because the ictal data of S2 displays similar seizure onset activities within each channel, and the EI method cannot tell the difference from either their timing orders or their energy strengths. The case of S2 suggests that when the ictal data cannot provide sufficient diagnostic information, the interictal data can be used to provide extra information for SOZ location, showing the essential value of interictal SEEG data analysis. **(B)** The ROC and AUC results of SOZ prediction, based on HI. Case S3 shows a low AUC of 0.49 based on HI but a high AUC of 0.99 based on EI. The HI results of S3 performed poorly because those false positive channels recorded plentiful high-frequency noises. EI and HI methods provide prediction results from different perspectives of views, so we recommended surgeons take a comprehensive consideration on both of them. **(C)** The HI results of S2 (marked with larger scales of contacts) and cortical reconstruction are displayed at the same time with clinically annotated SOZs (marked with red color).

**Figure 9:**
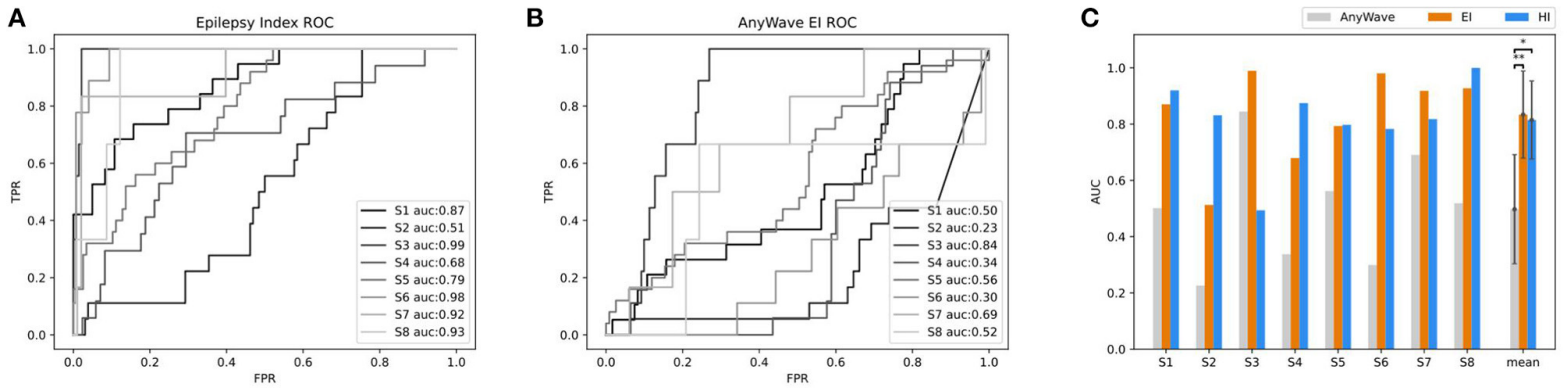
Comparisons between AnyWave EI method and the EI and HI of BrainQuake. **(A)** The ROC and AUC results of SOZ predictions, based on BrainQuake EI. **(B)** The ROC and AUC results of SOZ prediction, based on AnyWave EI. In most cases, BrainQuake EI shows a greater prediction effect than AnyWave EI. **(C)** Comparisons of AUC values between AnyWave EI and BrainQuake EI and HI. A Wilcoxon signed-rank test was performed between the prediction results of AnyWave and BrainQuake. The AUC of BrainQuake EI and HI are both significantly higher than that of AnyWave EI (*p* = 0.0078 and *p* = 0.0391, respectively, two-sided Wilcoxon signed-rank test, *p < 0.05, **p < 0.01).

To evaluate the accuracy of predicting SOZ using EI and HI methods, the selection of the clinician of the SOZ electrode contacts of patients was used as the ground truth. The receiver operator curve (ROC) and the corresponding area under the curve (AUC) were further used to evaluate the consistency between the index-based prediction and the clinical diagnosis. The average AUC of EI and HI on five patients are 0.83 and 0.80, respectively (with EI of S2 excluded) (Figures 8A,B). We could observe that on patient S1, both EI and HI have achieved excellent SOZ prediction results, which suggests a valid estimation of SOZs using both methods. The AUC value of S2 based on EI is close to 0.5 and has no predictive effect, due to the fact that the ictal data of S2 displays similar seizure onset activities within every single channel, and the EI method cannot tell the difference from either their timing orders or energy strengths. In contrast, the AUC of S2 based on interictal HI reaches 0.83, which is highly consistent with the clinically annotated SOZs. The case of S2 suggests that when the ictal data cannot provide sufficient diagnostic information, the interictal data can be used to provide extra information for SOZ location, showing the essential value of the interictal SEEG data analysis. In addition, the AUC value of S3 based on HI is 0.49, while its AUC based on HI reaches 0.99. The HI results of S3 performed poorly because those false-positive channels recorded plentiful high-frequency noises. The cases of S2 and S3 suggested the cross-reference value of EI and HI. Finally, we displayed SOZ predictions on reconstructed cortical volume for clinicians to verify the results with imaging evidence (Figure 8C). For the case of S2, we marked the clinically annotated contacts as larger spheres and the HI-based SOZs as red spheres, which shows consistency between these two groups. Moreover, we tried a similar EI module in a software, AnyWave (Colombet et al., 2015), to our ictal dataset, and it shows that the EI predictions of BrainQuake have higher ROCs in most cases (Figures 9A,B). The comparisons also show that the AUC of BrainQuake EI and HI both is significantly higher than that of AnyWave EI (*p* = 0.0078 and *p* = 0.0391, respectively, two-sided Wilcoxon signed-rank test, Figure 9C).

## Discussion and Conclusion

The intracranial SEEG data provide abundant electrophysiological information from the human brain for surgical planning and brain research. With the prevalence of SEEG recording in recent years, a large number of neurodata have been generated while researchers are exploring a way to make the best use of it. The challenge lies in both the fusion of multimodal neurodata and intensive computation during the SEEG analysis. In this study, we have introduced a self-sustained Python toolbox, i.e., BrainQuake, integrating multiple approaches to form a complete solution. For the structural data, the electrode module and the surface module provide fast and automated pipelines for surface reconstruction and electrode localization, with only raw MRI T1 and CT images needed for processing. For the functional data, both ictal and interictal modules exploit the long range of SEEG data and provide a presurgical estimation of SOZs. Blending structural and functional results, we provided neurosurgeons with a comprehensive tool for surgical planning. Neuroscientists who are using SEEG to study human brains will also be benefited from our toolbox.

The electrode localization approach implemented in BrainQuake divides the problem into two parts, namely, a global level of electrode clustering and a local level of contact segmentation. BrainQuake innovates in the level of automatic electrode voxel clustering. The semiautonomous methods require either additional input messages or a graphical user interface (GUI) to complete this process, i.e., the efficiency and user experience of which highly depends on the quality of images and preprocessing steps. Our algorithm, which is the combination of 3D Hough Transform and Gaussian Mixture Model, managed to take advantage of both geometric prior and graphical information embedded in CT images. The Hough Transform helps to detect the geometric characteristic of the objects in the image. Whatever the image resolution is high or low, electrode shafts are always straight and highlighted from the background. From this perspective, a pattern recognition algorithm can, in fact, be used to exploit the image instead of scanning it slice by slice. To our knowledge, this valid and useful geometric property has never been utilized in any other SEEG electrode localization method before. The Hough Transform makes electrode shafts be recognized automatically, although it may not return us a precise result. The recognized directions may deviate slightly from the shaft, or a recognized centroid may not be in the exact center of the actual electrode. However, the result can be much close to the true state, which is a good starting point for initializing the clustering algorithm. Thus, we removed the complicated manual intervention, that is, to replace the procedure of telling a software where the electrodes locate with automatic splicing of algorithms, and the pipeline consumes much less time than previous tools.

As for the subsequent step of contact segmentation for every single electrode, the algorithm of the center-of-mass convergence (Arnulfo et al., 2015) has shown interpretable principles and valid results. In our pipeline, we applied this algorithm to each electrode one by one after electrode clustering and acquire the final contact coordinates. We used axis-contact distance and adjacent contact distance error to estimate the geometric characteristics of the segmentation results. However, those two parameters are, in fact, the indirect ways of validating whether the contacts are properly located. Several factors may influence the error distributions. An electrode can bend slightly in the brain, in which case there is a possibility that fluctuations occur in the distributions of both parameters. It can generate some outliers in the distribution of axis-contact distance since the contacts are no longer scattered along a straight line and the deviations of contacts from the regressed line, in fact, exist. Moreover, due to the bending, the adjacent contact distance may shrink slightly as the contacts bear the force to be compressed to each other. Reflecting on Figure 7D, there are more distance errors lying in the negative half range than in the positive half range. In other cases, failures do exist due to the quality of the raw CT images. There are possibilities that the algorithm cannot find a local center-of-mass in a region and keep looking for highlights along the direction and finally converge to the next contact. This can explain the positive outliers in Figure 7D. We encountered a worse situation that the two regions of highlights were too close to each other and so the converging point just kept jumping from one optimal to another. We fixed this problem by implementing a counting index of convergence in the algorithm setting a forcing scheme to stop the infinite loop and choosing a voxel with higher voxel values just in case. We could notice that the design of the center-of-mass convergence algorithm does have its deficits and may not give us highly precise results. The recommended redeeming method is still visual checking. As for the essentiality of precise contact locations and then the locations of potential SOZs, one must not skip the procedure of manual checking. By projecting the contact results onto the registered CT image on a NIfTI image reading software such as “Freeview” (Figure 7B), we could go through the slices to check if the contacts recognized are matched with the highlighted voxels in the image. If an error is detected, surely one can erase a misplaced contact and add a new one by hand.

The automatic SOZ prediction methods usually use the onset order of high-frequency activity at each contact during the seizure or the specific distribution of abnormal activity during the interictal period as pathological features (Bartolomei et al., 2008; Barkmeier et al., 2012; Navarrete et al., 2016) These methods have already been integrated into some software independently (Tadel et al., 2011; Colombet et al., 2015). We tried a similar EI module in a software, AnyWave (Colombet et al., 2015), to our ictal dataset, and the comparison results show that the EI predictions of BrainQuake have higher ROCs in most of the cases (Figures 9A,B). Although the seizure data are considered to be more relevant to SOZ prediction, it may be difficult to capture or it may not provide enough information for the diagnosis, resulting in a relatively low AUC. Meanwhile, a large amount of interictal SEEG has not been fully utilized. The pathological information extracted from the long-term data may also have good predictive power on SOZ and is more immune to noises than the ictal data. As shown in our results (Figure 9C), HI derived from the interictal data is a good supplement to the EI method, and clinicians can compare the consistency between them. BrainQuake may serve as a platform for exploring the causal relationships between these two kinds of predictions and ultimately lead to better clinical diagnoses.

The processing of the long-term interictal data also gives rise to the challenge of computing power. The progress in deep learning has led to the development of high-performance parallel computing, and meanwhile, the acceleration capability of GPUs may be a solution to massive SEEG data and its high-load computing. At present, the mechanisms of seizures and interictal discharges are still unclear, and they may reflect different aspects of the epileptic network (Jiruska et al., 2017; Grinenko et al., 2018). In the future, we plan to implement a GPU module for the long-term interictal SEEG analysis in BrainQuake, and the prediction methods from the perspective of epileptic networks are to be explored.

BrainQuake is designed to be an auxiliary tool for epilepsy neurosurgeons and technicians, trying to convey a presurgical evaluation solution with blended functional and structural neurodata. Most current software or toolboxes focus on one or a few steps, developing splendid algorithms or techniques for data processing, but in clinical practice, it is a cumbersome task to merge all kinds of results into one system or coordinate. Also, several steps consume a lot of time and effort to do repeated work, resulting in an inefficient working procedure. BrainQuake commits to freeing surgeons and technicians from tedious and time-consuming work, allowing them to concentrate on the steps that rely more on common sense and medical expertise short in machine algorithms. In the upcoming era of big neurodata, this kind of human-computer synergy is an efficient approach to data utilization, and we believe that it will eventually promote the fields of both neurology and neuroscience.

## Data Availability Statement

The example dataset presented in this study can be found in https://doi.org/10.5281/zenodo.5675459. The codes can be found on Github (https://github.com/HongLabTHU/Brainquake).

## Ethics Statement

The studies involving human participants were reviewed and approved by Ethics Committee of Tsinghua Yuquan Hospital. Written informed consent to participate in this study was provided by the participants' legal guardian/next of kin.

## Author Contributions

BH, FC, and KW conceived the work, contributed to drafting, and revising the article. FC and KW designed the software. FC developed the surface module and the electrode module. KW and TZ developed the ictal module. KW developed the interictal module. HW and WZ collected the experimental data. All authors contributed to the article and approved the submitted version.

## Funding

This study was financially supported by the National Key R&D Program of China (2017YFA0205904 to BH).

## Conflict of Interest

The authors declare that the research was conducted in the absence of any commercial or financial relationships that could be construed as a potential conflict of interest.

## Publisher's Note

All claims expressed in this article are solely those of the authors and do not necessarily represent those of their affiliated organizations, or those of the publisher, the editors and the reviewers. Any product that may be evaluated in this article, or claim that may be made by its manufacturer, is not guaranteed or endorsed by the publisher.
